# Association of GNAS imprinting defects and deletions of chromosome 2 in two patients: clues explaining phenotypic heterogeneity in pseudohypoparathyroidism type 1B/iPPSD3

**DOI:** 10.1186/s13148-018-0607-8

**Published:** 2019-01-07

**Authors:** F. M. Elli, L. deSanctis, M.A. Maffini, P. Bordogna, D. Tessaris, A. Pirelli, M. Arosio, A. Linglart, G. Mantovani

**Affiliations:** 1 Fondazione IRCCS Ca’ Granda Ospedale Maggiore Policlinico, Endocrinology Unit, Milan, Italy; 2 Department of Clinical Sciences and Community Health, University of Milan, Milan, Italy; 3 Department of Public Health and Paediatric Sciences, University of Torino, Turin, Italy; 4 APHP, Paediatric Endocrinology and Diabetology for Children, Reference Centre for Rare Disorders of Calcium and Phosphate Metabolism, Platform of Expertise Paris-Sud for Rare Diseases and Filière OSCAR, Bicêtre Paris-Sud Hospital, 94270 Le Kremlin-Bicêtre, France; 5 APHP, Department of Endocrinology and Diabetology, Reference Centre for Rare Disorders of Calcium and Phosphate Metabolism, 94270 Le Kremlin-Bicêtre, France

**Keywords:** PHP-1B, iPPSD, 2q37, Modifier gene, GNAS, Methylation defect, AHO, Imprinting

## Abstract

**Background:**

The term pseudohypoparathyroidism (PHP) describes disorders derived from resistance to the parathyroid hormone. Albright hereditary osteodystrophy (AHO) is a disorder with several physical features that can occur alone or in association with PHP.

The subtype 1B, classically associated with resistance to PTH and TSH, derives from the epigenetic dysregulation of the GNAS locus. Patients showing features of AHO were described, but no explanation for such phenotypic heterogeneity is available.

An AHO-like phenotype was associated with the loss of genetic information stored in chromosome 2q37, making this genomic region an interesting object of study as it could contain modifier genes involved in the development of AHO features in patients with GNAS imprinting defects.

The present study aimed to screen a series of 65 patients affected with GNAS imprinting defects, with or without signs of AHO, for the presence of 2q37 deletions in order to find genes involved in the clinical variability.

**Results:**

The molecular investigations performed on our cohort of patients with GNAS imprinting defects identified two overlapping terminal deletions of the long arm of chromosome 2. The smaller deletion was of approximately 3 Mb and contained 38 genes, one or more of which is potentially involved in the clinical presentation. Patients with the deletions were both affected by a combination of the most pathognomic AHO-like features, brachydactyly, cognitive impairment and/or behavioural defects. Our results support the hypothesis that additional genetic factors besides GNAS methylation defects are involved in the development of a complex phenotype in the subgroup of patients showing signs of AHO.

**Conclusions:**

For the first time, the present work describes PHP patients with hormone resistance and AHO signs simultaneously affected by GNAS imprinting defects and 2q37 deletions. Although further studies are needed to confirm the cause of these two rare molecular alterations and to identify candidate genes, this finding provides novel interesting clues for the identification of factors involved in the still unexplained clinical variability observed in PHP1B.

**Electronic supplementary material:**

The online version of this article (10.1186/s13148-018-0607-8) contains supplementary material, which is available to authorized users.

## Background

The disorders related to parathyroid hormone (PTH) resistance and PTH signalling pathway impairment are historically named pseudohypoparathyroidism (PHP). The term Albright hereditary osteodystrophy (AHO) describes a clinical entity that could also associate with PHP, including several physical features such as brachydactyly, subcutaneous ossifications, round face, short stature, and obesity [1–3].

PHP is classified in different subtypes, PHP-1A, pseudo-PHP, PHP-1B, PHP-1C and PHP-2, according to the presence or absence of AHO, the in vivo response to exogenous PTH and the in vitro activity of the α subunit of the stimulatory G protein (Gsα), a key element of the cAMP signalling pathway encoded by *GNAS* [1–4]. However, this classification fails to differentiate all the patients due to the clinical and molecular overlap among the PHP subtypes and the recent characterization of diseases in the differential diagnosis of PHP. To clearly identify each different subtype and create a classification according to the molecular pathology, the EuroPHP network established a new classification to cover all the disorders of the PTH receptor and its signalling pathway, proposing the name inactivating PTH/PTH-related protein signalling disorder (iPPSD) [5, 6]. In particular, the iPPSD3 subtype includes methylation changes at one or more GNAS differentially methylated regions (DMRs), associated with or without a genetic (deletion) or cytogenetic (uniparental disomy (UPD)) defect.

Pseudohypoparathyroidism type 1B/iPPSD3 (MIM#603233) patients classically show resistance to PTH and TSH in the absence of additional clinical features, but in the past years, patients showing physical features of AHO have been described [7–12]. Several efforts were made to find a correlation between the degree of GNAS epigenetic defects and the severity of the disease in terms of age at diagnosis (as a marker of disease precocity), the number or degree of hormonal resistances and the number of AHO symptoms; however, no explanation for the phenotypic heterogeneity in iPPSD3 was found [11, 13, 14]. Among the factors that may explain the differences in disease expression and deserve investigation are modifier genes [15, 16].

Mutations in regions different from 20q were reported in small subsets of clinically diagnosed PHP patients with no detectable GNAS defects, highlighting the clinical overlap with diseases in the differential diagnosis of PHP: acrodysostosis (ACRDYS, *PRKAR1A* and *PDE4D* genes, MIM#101800 and #614316, respectively) and brachydactyly mental retardation syndrome (BDMR, also known as AHO-like syndrome or 2q37 microdeletion syndrome, MIM#600430) [5, 17].

From the molecular point of view, BDMR patients carry structural rearrangements with breakpoints at or within chromosome 2 region q37 [18]. Although BDMR is a contiguous gene deletion syndrome with a significantly variable clinical presentation, the hallmark reported in almost all the patients is an AHO-like phenotype characterized by mild to moderate intellectual disability/developmental delay, behavioural abnormalities, short stature, obesity, a characteristically dysmorphic face and brachydactyly type E [18].

The association of an AHO-like phenotype with the loss of genetic information stored in the 2q37 makes this genomic region interesting as it could contain modifier genes involved in the development of AHO features in iPPSD3 patients. Therefore, in the present study, we screened 65 patients affected with broad sporadic GNAS imprinting defects, with (*n* = 32) or without signs of AHO (*n* = 33), for the presence of BDMR-associated 2q37 deletions in order to find genes possibly involved in the phenotypic heterogeneity observed in iPPSD3.

## Results

**Fig. 1:**
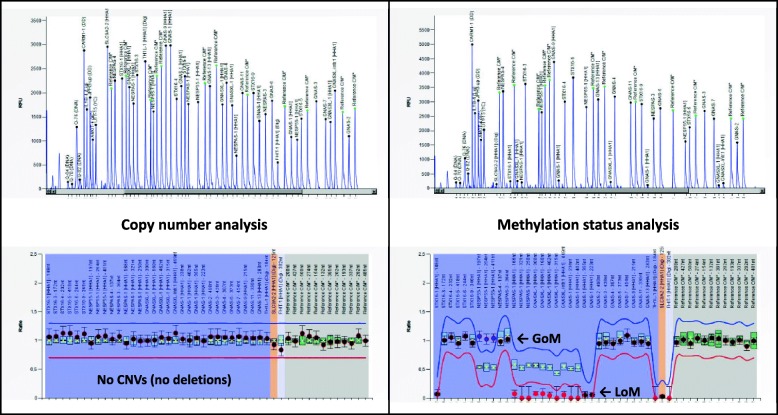
Representative plots of the MS-MLPA analysis by Coffalyser showing the LoI at the GNAS locus in iPPSD3 patients. Left panel: copy number analysis—data show a normal bialleleic status of the STX16 and GNAS regions, which means the absebce of any no structural defect (in particular no deletions). Right panel: methylation analysis—data show a broad loss of imprinting at the GNAS locus. Calculated ratios are reported on the *Y*-axis and probes on the *X*-axis. Red dots highlight the LoM (meth ratio < 0.5) and blue dots the GoM (meth ratio > 0.5)

All the PHP patients were affected by sporadic GNAS methylation alterations involving all the differentially methylated regions (gain of methylation, GoM, at the NESP DMR and loss of methylation, LoM, at the AS, XL and AB DMRs) (Fig. 1). We excluded a 20q UPD in all the cases.

**Fig. 2:**
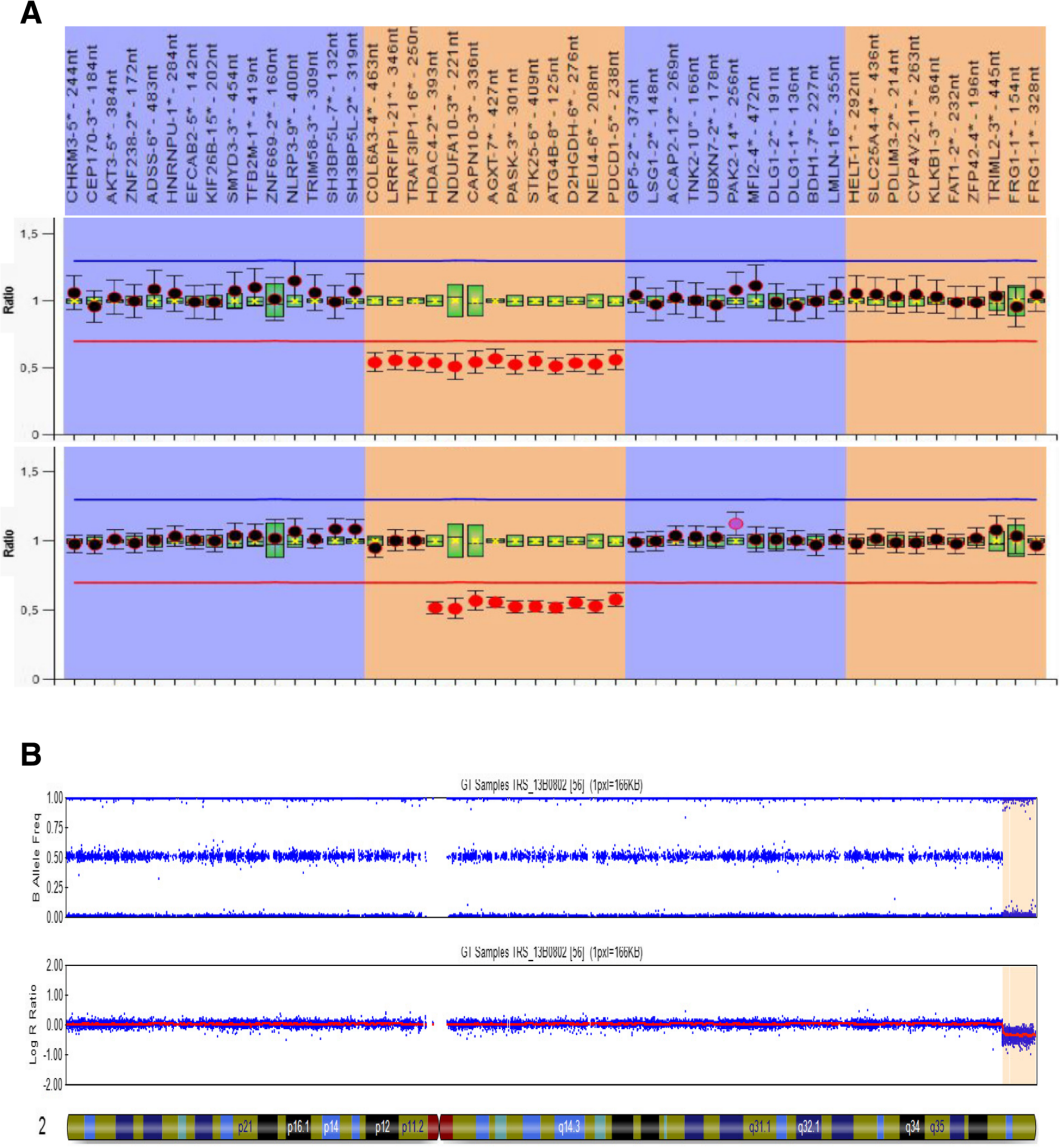
**a** Plots of the MLPA analysis by Coffalyser showing the 2q37 deletions found in our cohort of iPPSD3 patients. Upper panel: patient 4, with a deletion removing genes from *COL6A3* to *PDCD1*. Lower panel: patient 3, with a deletion removing genes from *HDAC4* to *PDCD1*. Calculated ratios are reported on the *Y*-axis and probes on the *X*-axis. Red dots highlight the heterozygous deletion (allelic loss < 0.5). **b** Plot of the array analysis performed in patient 4 confirming a 8.2 Mb subtelomeric terminal deletion of the long arm of the chromosome 2 (hg19 2:234,782,915—243,029,273)

The MLPA analysis identified two deletions that include the 2q37 cytoband associated with BDMR. In particular, the assay showed a 4.5 Mb deletion from the *COL6A3* gene to the *PDCD1* gene in patient 4 and a 2.5 Mb deletion from the *HDAC4* gene to the *PDCD1* gene in patient 3 (Fig. 2a and Additional file 1: Table S2). Due to the limits of the MLPA technique, point mutations and defects outside genomic sequences complementary to the MLPA probes were not detected. We could not exclude the presence of genetic alterations different from CNVs in the other patients. Molecular karyotyping is needed to precisely determine the extent of a deletion; thus, we performed the array analysis for patient 4, and we confirmed an 8.2 Mb subtelomeric terminal 2q deletion, 46,XX;arr[hg19]2q37.1q37.3(234,782,915–243,029,273)x1 (Fig. 2b).

The qualitative and semiquantitative analysis of 2q37 VNTRs confirmed the rearrangements detected by MLPA and allowed prediction of the extension of these genetic defects with all the detected heterozygous VNTRs representing a biallelic condition (Additional file 2: Figure S1 and Additional file 1: Table S2). The semiquantitative PCR analysis showed a signal reduction of approximately 50% compared to that of the normal controls for the monoallelic regions, reflecting the loss of the deleted allele.

**Fig. 3:**
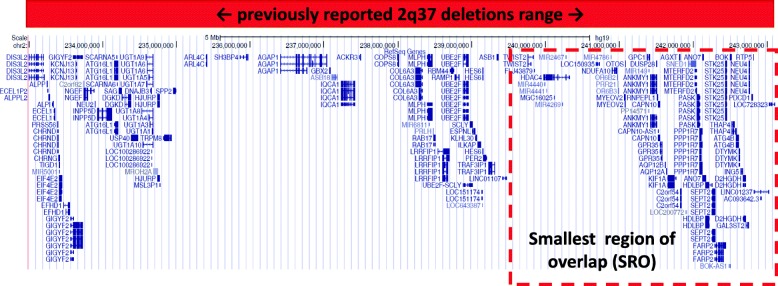
Representative figure from the UCSC Genome Browser resuming the extension range of previously reported BDMR-associated deletions and genes included. The smallest region of overlap (SRO) between known and our deletions is highlighted in red

Because both deletions were terminal, we were not able to identify the precise upper breakpoint locations; however, by merging the data obtained from different techniques, we determined the approximate size of the defects and the number of deleted genes: 8,416,458–9,491,396 bps/55 genes for patient 4 and 2,924,948–3,893,174 bps/38 genes for patient 3 (Fig. 3 and Additional file 1: Table S2).

The smaller deletion detected in patient 3, which overlapped with that found in patient 4 and differs only in the upper breakpoint, represented the smallest region of overlap (SRO) containing the potential gene or genes involved in the clinical presentation (Fig. 3 and Additional file 1: Table S2).

Patient 4 presented resistance to PTH, brachydactyly, psychomotor retardation, gastroesophageal and vesicoureteral reflux, celiac disease and subluxation of the temporomandibular joint. The patient’s mother showed signs of brachydactyly, which strengthens the idea of inherited factors associated with specific clinical features. Patient 3, in addition to resistance to PTH and TSH, showed brachydactyly, neuro-psychomotor retardation (IQ = 68–70%, need for extra help in school), stature growth slowing (less than the 25th percentile), strabismus and scoliosis.

According to the hypothesis of additional genetic factors being involved in the development of a complex phenotype in the subgroup of iPPSD3 patients with signs of AHO, both patients were affected by a combination of the most pathognomic AHO-like features, which are brachydactyly and cognitive impairment and/or behavioural defects. 

## Discussion

The sporadic iPPSD3 is an imprinting disorder characterized by methylation abnormalities at multiple GNAS DMRs. Patients are resistant to the action of PTH and TSH in the absence of additional clinical features, although a subset of patients also presents physical features of Albright hereditary osteodystrophy [8–12]. Several factors may underlie such differences in disease expression including modifier genes. To date, no explanation has been found for the apparent absence of (epi)genotype-phenotype correlations, and the search for possible causes is still underway [11, 12, 15, 16].

Recently, different research groups have reported molecular defects in regions different from 20q in small subsets of clinically diagnosed PHP patients with no detectable GNAS defects, further emphasizing the clinical overlap with diseases in differential diagnosis such as the BDMR [5, 17].

BDMR, initially called AHO-like syndrome, derives from the loss of genes included in the 2q37 region; however, the large extension of known deletions has no clearly defined candidate genes or established genotype-phenotype correlations [18].

The experience gained in studying the molecular mechanisms that explain the phenotypic heterogeneity in iPPSD3 led us to hypothesize the involvement of additional loci besides GNAS, and the best candidate region for screening was the 2q37 region. Thus, we investigated this genomic region in a cohort of 65 sporadic iPPSD3 patients (32 had signs of AHO and 33 did not) to determine the existence of a regulatory and/or functional link between GNAS and the 2q37 region.

We found 2 different 2q37 deletions in 2 unrelated subjects displaying signs of AHO that overlapped with defects already associated with BDMR and included several genes proposed to be responsible for the AHO-like phenotype. Admittedly, limitations of this study were that we could not perform the aCGH analysis for patient 3 to identify the precise size of the deletion and we did not analyse parental samples to investigate the inheritance and pathogenicity.

For appropriate genetic counselling, patients should be made aware that 2q37 deletions are inheritable genetic defects with a 50% recurrence risk in the offspring. However, it is not currently possible to predict whether such defects would associate with a clinical phenotype.

Several attempts to find a clear relationship between phenotypic features and the minimal deletion interval have been performed, but the clinical variability prevented the obtaining of useful predictors of clinical presentation [19, 20]. It must also be kept in mind that the syndromic phenotype of 2q37 deletion patients might result from a contiguous gene deletion syndrome.

We selected potential candidate genes by localizing the smallest region of overlap, which was a 3.8 Mb sequence containing 38 genes, and we compared it with 2q37 deletions reported in the literature. This region, described by Chassaing and Shrimpton (patient R.V.W.), is the smallest region characterized in BDMR patients [21, 22]. The common clinical features reported in these two BDMR patients were an uneventful prenatal and birth history, a normal hormone profile, delayed cognitive impairment/moderate mental retardation, obesity, mild facial dysmorphic features and brachydactyly. Our patients presented, in addition to the hormone resistance classically associated with iPPSD3 and typically absent in BDMR, brachydactyly and neuro-psychomotor impairment. Moreover, similar to patient R.V.W., patient 3 also had short stature and a dysmorphic face [21, 22].

According to their known function, *HDAC4*, *PASK*, *FARP2* and *STK25* were confirmed as the most promising candidate genes [21–25]. The histone deacetylase 4 (*HDAC4*, MIM*605314) is a corepressor for DNA-binding transcription factors, playing a pivotal role in myogenesis, skeletal development and neurogenesis [24, 25]. The PAS kinase (*PASK*, MIM* 607505) is an evolutionarily conserved protein that regulates the function of many intracellular signalling pathways in response to both extrinsic and intrinsic stimuli [23]. The FERM, ARHGEF, and pleckstrin domain-containing protein 2 (FARP2, MIM* 617586) functions as a guanine nucleotide exchange factor (GEF) for Rho family small GTPases and plays a role in regulating the actin cytoskeleton [23]. The Serine/Threonine protein kinase 25 (STK25, MIM* 602255) is a member of the Ste20/PAK family implicated in heterotrimeric G protein signalling [21, 22].

These data suggested that one or more genes essential for skeletal morphogenesis and neurodevelopment were located in the SRO. Such genes could encode proteins acting through a Gsα-mediated transduction pathway without affecting those activated by other mediators.

Both patients described in the present manuscript displayed signs of AHO in addition to the endocrine characteristics of PTH resistance. Interestingly, hormone resistance is usually absent in BDMR, although one patient with an AHO-like phenotype and raised levels of PTH was described by Power and colleagues in 1997 [26]. No further comparison with the data from the literature could be performed since the BDMR patients were not systematically evaluated for all the symptoms, in particular for the endocrine function and the presence of GNAS imprinting defects.

Imprinting disorders, such as transient neonatal diabetes mellitus, have been associated with point mutations in genes encoding factors involved in the establishment and/or maintenance of imprinting [27, 28]. It is unclear whether imprinting defects causing the sporadic form of iPPSD3 are secondary to the genetic defects. Due to the large extension of the 2q37 deletion and the high content in transcripts with an unknown function, this region could host genes involved in GNAS epigenetic regulation and should be considered in future studies.

In conclusion, we first described the existence of iPPSD3 patients with hormone resistance and AHO signs simultaneously affected by GNAS imprinting defects and 2q37 deletions. The discovery of these complex genotypes highlighted the difficulty in the interpretation of genetic testing in the presence of a combination of rare molecular alterations in the same individual. The screening of 2q37 deletions is already indicated in the molecular diagnostic setting of patients who show several features of AHO but have normal Gsα levels and no endocrine abnormalities. In our opinion, it is premature to propose the search for 2q37 alterations in patients with iPPSD3 for diagnostic purposes since only two affected patients have been identified so far. Further studies to confirm a causality between GNAS and 2q37 defects and to identify novel candidate genes are needed. Nevertheless, considering approximately 20% of patients with GNAS epigenetic defects have hormone resistance with AHO signs [29], our findings provide interesting clues for the identification of factors involved in the still unexplained clinical variability observed in iPPSD3.

## Materials and methods

The study included 65 patients with a clinical diagnosis of a sporadic iPPSD3. Most patients have been reported in our previous work to investigate the presence of a correlation between GNAS epigenetic alterations and clinical characteristics in patients with pseudohypoparathyroidism type 1 [30].

The clinical diagnosis of PHP was based upon the presence of PTH resistance, i.e., hypocalcaemia, hyperphosphatemia and raised serum PTH levels in the absence of vitamin D deficiency and renal insufficiency. Twenty-five out of the 65 patients presented with TSH resistance, documented by raised TSH serum levels (TSH > 3.9 mU/L) in the absence of anti-thyroid antibodies and the presence of a normal thyroid scan (data not shown).

Thirty-two patients showed at least 1 and up to 4 typical AHO manifestations, which include brachydactyly (*n* = 13, shortening of the fourth and/or fifth metacarpals defined as the metacarpal sign and/or shortening below − 2SDS at the metacarpophalangeal profile pattern in at least one metacarpal bone or distal phalanx), short stature (*n* = 10, height below the 3rd percentile for chronological age), obesity (*n* = 15, BMI > 30 kg/m^2^ in adults and weight > 97th percentile in children), round face (*n* = 17), and cognitive impairment and/or behavioural defects (*n*= 9). Only two subjects of this group showed both brachydactyly and mental/behavioural defects, which are clinical manifestations associated with BDMR.

The clinical details are shown in Additional file 1: Table S1. Informed consent for genetic and epigenetic studies was obtained from all the subjects involved in the study.

The molecular investigations were performed on the genomic DNA extracted from peripheral blood (Nucleon BACC2 genomic DNA purification kit, GE Healthcare, Piscataway, NJ, USA). The direct sequencing of *GNAS* coding exons and flanking intronic sequences (ENSEMBL ID: ENSG00000087460) and the methylation-specific-multiplex ligand-dependent probe amplification (MS-MLPA, ME031 vB1-1012 GNAS probemix by MRC-Holland, Amsterdam, The Netherlands) analysis were performed as previously described [30, 31].

Informative genetic markers, such as variable number tandem repeats (VNTRs) in the 20q region, were evaluated by capillary electrophoresis of fluorescent-labelled PCR amplicons (ABI3130xl Genetic Analyzer, Applied Biosystems, Foster City, CA) to exclude the presence of uniparental disomy (UPD) [32].

The presence of copy number variants (CNVs) affecting the 2q37 region was detected by MLPA using the P264-Human Telomere-9 vB1-0514 (MRC-Holland, Amsterdam, The Netherlands). The data analysis was carried out using the Coffalyser software (MRC-Holland, Amsterdam, The Netherlands).

For the 2q37 VNTRs genotyping (for a complete list of tested VNTRs see the Additional file 1: Table S2), we set up a cost-effective three-primer approach based on the simultaneous use of a couple of sequence-specific primers, where the reverse primer containing a poly-A tail at the 5′ end to allow easier allele scoring, associated with a fluorescently labelled universal forward M13 tail FAM oligonucleotide. All primers and experimental conditions are available upon request.

Additionally, in 1 patient bearing the 2q37 deletion, a genome-wide genotyping array was performed using the HumanCytoSNP-12 (Illumina, San Diego, California, USA). A panel designed for the analysis of genetic and structural variations that are most relevant to human disease and data analysis was performed with the BlueFuse Multi Software v4.4.

## Additional files

Additional file 1: **Table S1.** Clinical characteristics and molecular analysis of patients included in the present study. Abbreviations and symbols: ID, identification number; F, female; M, male; ↑, value not available but reported as above reference range; ↔, value not available but reported as in range; ↓, value not available but reported as below reference range; SS, short stature; Ob, obesity; RF, round/dysmorfic facies; Br, brachydactyly; MR, mental retardation and/or behavioural defects; OS, ectopic ossification. The molecular diagnosis age is reported in years. Biochemical values outside the normal range are highlighted in bold. For PTH, the normal range is 10–65 pm/mL; for TSH, the normal range is 0.4–3.9 mU/L; for calcemia, the normal range is 9.0–10.5 mg/dL; for phoshpatemia, the normal range is 2.8–4.5 mg/dL. **Table S2.** Table resuming analysed variable number tandem repeats (VNTR), MLPA probes and gene location in the 2q37 region of deleted patients. Abbreviations: het, heterozygous; homo, homozygous; del, deleted. (DOCX 47 kb)

Additional file 2: **Figure S1.** 2q37 marker analysis in iPPSD3 deleted patients for confirm and characterize found structural rearrangements. Selection of homozygous (1 peak) and heterozygous (2 peaks) VNTRs from patients 3 (right panels) and 4 (left panels). (PPTX 4081 kb)

## Abbreviations

ACRDYS: Acrodysostosis
AHO: Albright hereditary osteodystrophy
BDMR: Brachydactyly mental retardation syndrome
DMR: Differentially methylated region
Gsα: Alpha subunit of the stimulatory G protein
iPPSD: Inactivating PTH/PTH related protein
MIM: Mendelian inheritance in man
MLPA: Multiplex ligand-dependent probe amplification
MS-MLPA: Methylation sensitive multiplex ligand-dependent probe amplification
PHP: Pseudohypoparathyroidism
PHP-1A: Pseudohypoparathyroidism type 1A
PHP-1B: Pseudohypoparathyroidism type 1B
PHP-1C: Pseudohypoparathyroidism type 1C
PHP-2: Pseudohypoparathyroidism type 2
PPHP: Pseudopseudohypoparathyroidism
PTH: Parathyroid hormone
SRO: Smallest region of overlap
TSH: Thyroid stimulating hormone
UPD: Uniparental disomy
VNTR: Variable number tandem repeat
