# Supplementary material for: Association of GNAS imprinting defects and deletions of chromosome 2 in two patients: clues explaining phenotypic heterogeneity in pseudohypoparathyroidism type 1B/iPPSD3
Source: Clin Epigenetics. 2019 Jan 7;11:3. doi: 10.1186/s13148-018-0607-8 (PMC6322333; doi:10.1186/s13148-018-0607-8)
Supplement: Supplementary file 1 — Table S1. Clinical characteristics and molecular analysis of patients included in the present study. Abbreviations and symbols: ID, identification number; F, female; M, male; ↑, value not available but reported as above reference range; ↔, value not available but reported as in range; ↓, value not available but reported as below reference range; SS, short stature; Ob, obesity; RF, round/dysmorfic facies; Br, brachydactyly; MR, mental retardation and/or behavioural defects; OS, ectopic ossification. The molecular diagnosis age is reported in years. Biochemical values outside the normal range are highlighted in bold. For PTH, the normal range is 10–65 pm/mL; for TSH, the normal range is 0.4–3.9 mU/L; for calcemia, the normal range is 9.0–10.5 mg/dL; for phoshpatemia, the normal range is 2.8–4.5 mg/dL. Table S2. Table resuming analysed variable number tandem repeats (VNTR), MLPA probes and gene location in the 2q37 region of deleted patients. Abbreviations: het, heterozygous; homo, homozygous; del, deleted. (DOCX 47 kb) [file 13148_2018_607_MOESM1_ESM.docx]

**Table S1: Clinical characteristics and molecular analysis of patients included in the present study**. Abbreviations and symbols: ID, identification number; F, female; M, male; ↑, value not available but reported as above reference range; ↔, value not available but reported as in range; ↓, value not available but reported as below reference range; SS, short stature; Ob, obesity; RF, round/dysmorfic facies; Br, brachydactyly; MR, mental retardation and/or behavioral defects; OS, ectopic ossification. The molecular diagnosis age is reported in years. Biochemical values outside the normal range are highlighted in bold. For PTH, the normal range is 10-65 pm/mL; for TSH, the normal range is 0.4-3.9 mU/L; for calcemia, the normal range is 9.0-10.5 mg/dL; for phoshpatemia, the normal range is 2.8-4.5 mg/dL.

| **pt ID** | **gender** | **PHP diagnosis age** | **PTH** | **TSH** | **Ca** | **P** | **rPTH** | **rTSH** | **SS** | **Ob** | **RF** | **Br** | **MR** | **OS** | **2q37del analysis** |
| --- | --- | --- | --- | --- | --- | --- | --- | --- | --- | --- | --- | --- | --- | --- | --- |
| **1** | M | 7 | **102** | **10** | 9 | 3.8 | X | X |  | X | X | X |  |  | WT |
| **2** | F | 29 | **534** | 2.11 | **7.5** | **6.8** | X |  | X | X | X | X |  |  | WT |
| **3** | M | 9 | **634** | **6.1** | **6.6** | **7.1** | X | X |  |  | X | X | X |  | del 2q |
| **4** | F | 15 | **188** | 2.2 | **7.8** | **5.9** | X |  |  |  |  | X | X |  | del 2q |
| **5** | F | 3 | **162** | 20 | **7.2** | **6.1** | X | X |  |  |  |  |  |  | WT |
| **6** | M | 10 | **382** | ↔ | **4.5** | **6** | X |  |  | X |  |  | X |  | WT |
| **7** | F | 31 | **806** | ↔ | **2.06** | **↓** | X |  | X |  | X |  | X |  | WT |
| **8** | M | 18 | **↑** | ↔ | **4.4** | **↓** | X |  |  |  | X |  | X |  | WT |
| **9** | M | 9 | **299** | ↔ | 10.2 | **7.5** | X |  |  |  |  |  | X |  | WT |
| **10** | F | 27 | **308** | **7.2** | **5.5** | **5.1** | X | X |  |  |  |  | X |  | WT |
| **11** | M | 10 | **1298** | **4.47** | **8.1** | **6.3** | X | X |  |  |  |  | X |  | WT |
| **12** | F | 4 | **890** | **5.04** | **7.2** | **8.3** | X | X |  |  |  |  | X |  | WT |
| **13** | M | 10 | **529** | 2.1 | **6.9** | **7.4** | X |  |  | X | X | X |  |  | WT |
| **14** | F | 68 | **↑** | ↔ | **↑** | **↓** | X |  | X | X |  | X |  |  | WT |
| **15** | F | 23 | **790** | 2.4 | **6.85** | **7.15** | X |  |  |  | X |  |  |  | WT |
| **16** | M | 37 | **233** | ↔ | **6.2** | **4.89** | X |  | X |  | X | X |  |  | WT |
| **17** | M | 12 | **606** | ↔ | **8.3** | **↓** | X |  | X |  | X | X |  |  | WT |
| **18** | F | 12 | **977** | **4.8** | **6.9** | **8.2** | X | X |  |  | X | X |  |  | WT |
| **19** | F | 31 | **264** | **5.58** | **6.5** | **6.9** | X | X |  | X |  | X |  |  | WT |
| **20** | M | 35 | **128** | 3.8 | **4.9** | **4.6** | X |  |  |  |  | X |  |  | WT |
| **21** | F | 7 | **1270** | 3.5 | **6.4** | **6.1** | X |  |  |  |  | X |  |  | WT |
| **22** | F | 28 | **317** | 2.9 | **7.4** | 3.8 | X |  | X | X | X |  |  |  | WT |
| **23** | M | 34 | **125** | **5.6** | **4.76** | **5.71** | X | X | X | X | X |  |  |  | WT |
| **24** | M | 20 | **338** | 2.1 | **6** | **5.5** | X |  |  | X |  |  |  |  | WT |
| **25** | F | 4 | **490** | 2.1 | **5.1** | **7.7** | X |  |  | X | X |  |  |  | WT |
| **26** | F | 17 | **333** | 3.3 | **7.6** | **4.8** | X |  |  | X | X |  |  |  | WT |
| **27** | M | 15 | **↑** | ↔ | **↑** | **↓** | X | X | X | X |  |  |  |  | WT |
| **28** | F | 5 | **437** | **5.8** | **3.8** | **7.8** | X | X |  | X |  | X |  |  | WT |
| **29** | M | 24 | **206** | 3.2 | **5.2** | **9.5** | X |  | X | X |  |  |  |  | WT |
| **30** | M | 16 | **468** | 2.3 | **8** | 3.6 | X |  |  | X | X |  |  |  | WT |
| **31** | F | 36 | **615** | 1.8 | **4.8** | **8.5** | X |  |  |  | X |  |  |  | WT |
| **32** | F | 13 | **647** | **6.97** | **5.53** | **9.29** | X | X | X |  |  |  |  |  | WT |
| **33** | F | 39 | **322** | 2.5 | **7.9** | 4.2 | X |  |  |  |  |  |  |  | WT |
| **34** | F | 39 | **436** | ↔ | **5.9** | **5.5** | X |  |  |  |  |  |  |  | WT |
| **35** | F | 38 | **210** | **8.6** | 9.5 | ↔ | X | X |  |  |  |  |  |  | WT |
| **36** | M | 22 | **454** | **4.9** | **8.3** | 4.3 | X | X |  |  |  |  |  |  | WT |
| **37** | M | 2 | **246** | **14.5** | **7.1** | 5.2 | X | X |  |  |  |  |  |  | WT |
| **38** | F | 14 | **188** | **4** | **6.9** | **5.4** | X | X |  |  |  |  |  |  | WT |
| **39** | M | 16 | **561** | 3.2 | **5.2** | **11.3** | X |  |  |  |  |  |  |  | WT |
| **40** | M | 26 | **315** | 2 | **6.4** | **6.5** | X |  |  |  |  |  |  |  | WT |
| **41** | F | 53 | **180** | **4.47** | **6** | **5.52** | X | X |  |  |  |  |  |  | WT |
| **42** | M | 32 | **114** | **5.4** | **8.5** | 4.3 | X | X |  |  |  |  |  |  | WT |
| **43** | M | 10 | **↑** | ↔ | **↑** | **↓** | X |  |  |  |  |  |  |  | WT |
| **44** | M | 18 | **288** | **7** | **5** | **7.7** | X | X |  |  |  |  |  |  | WT |
| **45** | M | 16 | **486** | 3.54 | **7.84** | **6.75** | X |  |  |  |  |  |  |  | WT |
| **46** | F | 9 | **1234** | **4.89** | **5.5** | **7.7** | X | X |  |  |  |  |  |  | WT |
| **47** | F | 27 | **425** | **6.81** | **5.1** | **6** | X | X |  |  |  |  |  |  | WT |
| **48** | F | 19 | **7.3** | ↔ | **5.62** | **6.68** | X |  |  |  |  |  |  |  | WT |
| **49** | F | 13 | **296** | ↔ | **7.79** | **4.87** | X |  |  |  |  |  |  |  | WT |
| **50** | F | 20 | **532** | 1.4 | **7.4** | 3.6 | X |  |  |  |  |  |  |  | WT |
| **51** | M | 12 | **899** | 2.65 | **5.81** | **7.5** | X |  |  |  |  |  |  |  | WT |
| **52** | F | 47 | **316** | 0.97 | **8.2** | **↓** | X |  |  |  |  |  |  |  | WT |
| **53** | M | 38 | **299** | **6.7** | **8.5** | 3.8 | X | X |  |  |  |  |  |  | WT |
| **54** | F | 27 | **1100** | 0.8 | **7** | **6.2** | X |  |  |  |  |  |  |  | WT |
| **55** | F | 10 | **↑** | ↔ | **↑** | **↓** | X |  |  |  |  |  |  |  | WT |
| **56** | M | 14 | **127** | **4.66** | **4.52** | **10.26** | X | X |  |  |  |  |  |  | WT |
| **57** | F | 33 | **125** | 1.47 | **5.3** | **5.3** | X |  |  |  |  |  |  |  | WT |
| **58** | M | 38 | **258** | **4.1** | **8.8** | 4.1 | X | X |  |  |  |  |  |  | WT |
| **59** | M | 22 | **205** | 3 | **7.9** | 3 | X |  |  |  |  |  |  |  | WT |
| **60** | F | 37 | **364** | 1.23 | **8** | 3.6 | X |  |  |  |  |  |  |  | WT |
| **61** | M | 42 | **160** | **7** | **6.7** | 4.5 | X | X |  |  |  |  |  |  | WT |
| **62** | F | 13 | **215** | 2.5 | **4.9** | **7.6** | X |  |  |  |  |  |  |  | WT |
| **63** | M | 1 | ↔ | ↔ | ↔ | ↔ | X |  |  |  |  |  |  |  | WT |
| **64** | F | 39 | **267** | 2.8 | **8.1** | 3.4 | X |  |  |  |  |  |  |  | WT |
| **65** | F | 12 | **347** | **4.5** | 9.2 | **6.3** | X | X |  |  | X |  |  |  | WT |

**Table S2: Table resuming analyzed variable number tandem repeats (VNTR), MLPA probes and gene location in the 2q37 region of deleted patients. Abbreviations: het, heterozygous; homo, homozygous; del, deleted.**

| **type** | **marker/probe ID** | **gene** | **Pt 4** | **Pt 3** |
| --- | --- | --- | --- | --- |
| **VNTR** | **D2S206** |  | **HET** | **HET** |
| **VNTR** | **D2S2205** |  | **HOMO** | **HOMO** |
| **VNTR** | **D2S336** |  | **HOMO** | **HET** |
| **VNTR** | **D2S2202** |  | **HOMO** | **HET** |
| **VNTR** | **D2S338** |  | **HOMO** | **HET** |
| **gene** |  | **RDC1/ACKR3** |  |  |
| **VNTR** | **D2S345** |  | **HOMO** | **HET** |
| **VNTR** | **D2S2968** |  | **HOMO** | **HET** |
| **MLPA/gene** | **09038-L09292** | **COL6A3** | **DEL** | **NO DEL** |
| **MLPA/gene** | **09037-L09291** | **LRRFIP1** | **DEL** | **NO DEL** |
| **VNTR** | **D2S1833** |  | **HOMO** | **HOMO** |
| **gene** |  | **RBM44** |  |  |
| **gene** |  | **RAMP1** |  |  |
| **gene** |  | **UBE2F** |  |  |
| **gene** |  | **SCLY** |  |  |
| **gene** |  | **ESPNL** |  |  |
| **gene** |  | **KLHL30** |  |  |
| **gene** |  | **FAM132B** |  |  |
| **VNTR** | **D2S2338** |  | **HOMO** | **HOMO** |
| **gene** |  | **ILKAP** |  |  |
| **gene** |  | **HES6** |  |  |
| **gene** |  | **PER2** |  |  |
| **gene** |  | **MIPT3/TRAF3IP1** |  |  |
| **MLPA/gene** | **09036-L13880** | **TRAF3IP1** | **DEL** | **NO DEL** |
| **gene** | **ASB1** | **ASB1** |  |  |
| **gene** | **TWIST2** | **TWIST2** |  |  |
| **MLPA/gene** | **10036-L11449** | **HDAC4** | **DEL** | **DEL** |
| **MLPA/gene** | **09034-L09288** | **NDUFA10** | **DEL** | **DEL** |
| **gene** |  | **OR6B2** |  |  |
| **gene** |  | **PRR21** |  |  |
| **gene** |  | **OR6B3** |  |  |
| **gene** |  | **MYEOV2** |  |  |
| **gene** |  | **OTOS** |  |  |
| **VNTR** | **D2S125** |  | **HOMO** | **HOMO** |
| **gene** |  | **GPC1** |  |  |
| **gene** |  | **ANKMY1** |  |  |
| **gene** |  | **DUSP28** |  |  |
| **gene** |  | **RNPEPL1** |  |  |
| **MLPA/gene** | **15667-L17633** | **CAPN10** | **DEL** | **DEL** |
| **gene** |  | **GPR35** |  |  |
| **gene** |  | **AQP12B** |  |  |
| **gene** |  | **AQP12A** |  |  |
| **gene** | **ATSV** | **KIF1A** |  |  |
| **MLPA/gene** | **10035-L11450** | **AGXT** | **DEL** | **DEL** |
| **gene** |  | **SNED1** |  |  |
| **gene** |  | **MTERFD2** |  |  |
| **MLPA/gene** | **09031-L09285** | **PASK** | **DEL** | **DEL** |
| **gene** | **PPP1R7** | **PPP1R7** |  |  |
| **gene** |  | **ANO7** |  |  |
| **VNTR** | **RH101** |  | **HOMO** | **HOMO** |
| **VNTR** | **RH104464** |  | **HOMO** | **HOMO** |
| **gene** | **HDLBP** | **HDLBP** |  |  |
| **gene** | **NEDD5** | **SEPT2** |  |  |
| **gene** | **FARP2** | **FARP2** |  |  |
| **VNTR** | **SHGC-32276** |  | **HOMO** | **HOMO** |
| **MLPA/gene** | **10034-L10544** | **STK25** | **DEL** | **DEL** |
| **gene** |  | **BOK** |  |  |
| **gene** |  | **THAP4** |  |  |
| **MLPA/gene** | **02782-L02224** | **ATG4B** | **DEL** | **DEL** |
| **gene** |  | **DTYMK** |  |  |
| **gene** |  | **ING5** |  |  |
| **MLPA/gene** | **15666-L17632** | **D2HGDH** | **DEL** | **DEL** |
| **gene** |  | **GAL3ST2** |  |  |
| **MLPA/gene** | **09029-L09283** | **NEU4** | **DEL** | **DEL** |
| **MLPA/gene** | **15664-L17629** | **PDCD1** | **DEL** | **DEL** |
| **gene** |  | **CXXC11** |  |  |
| **VNTR** | **D2S2985** |  | **HOMO** | **HOMO** |
| **VNTR** | **D2S2988** |  | **HOMO** | **HOMO** |
| **VNTR** | **D2S2986** |  | **HOMO** | **HOMO** |
| **VNTR** | **D2S447** |  | **HOMO** | **HOMO** |
| **VNTR** | **D2S2585** |  | **HOMO** | **HOMO** |
| **gene** |  | **CICP10** |  |  |
| **gene** |  | **ABC7** |  |  |
