# Supplementary material for: Association of GNAS imprinting defects and deletions of chromosome 2 in two patients: clues explaining phenotypic heterogeneity in pseudohypoparathyroidism type 1B/iPPSD3
Source: Clin Epigenetics. 2019 Jan 7;11:3. doi: 10.1186/s13148-018-0607-8 (PMC6322333; doi:10.1186/s13148-018-0607-8)
Supplement: Supplementary file 2 — Figure S1. 2q37 marker analysis in iPPSD3 deleted patients for confirm and characterize found structural rearrangements. Selection of homozygous (1 peak) and heterozygous (2 peaks) VNTRs from patients 3 (right panels) and 4 (left panels). (PPTX 4081 kb) [file 13148_2018_607_MOESM2_ESM.pptx]

## Slide 1
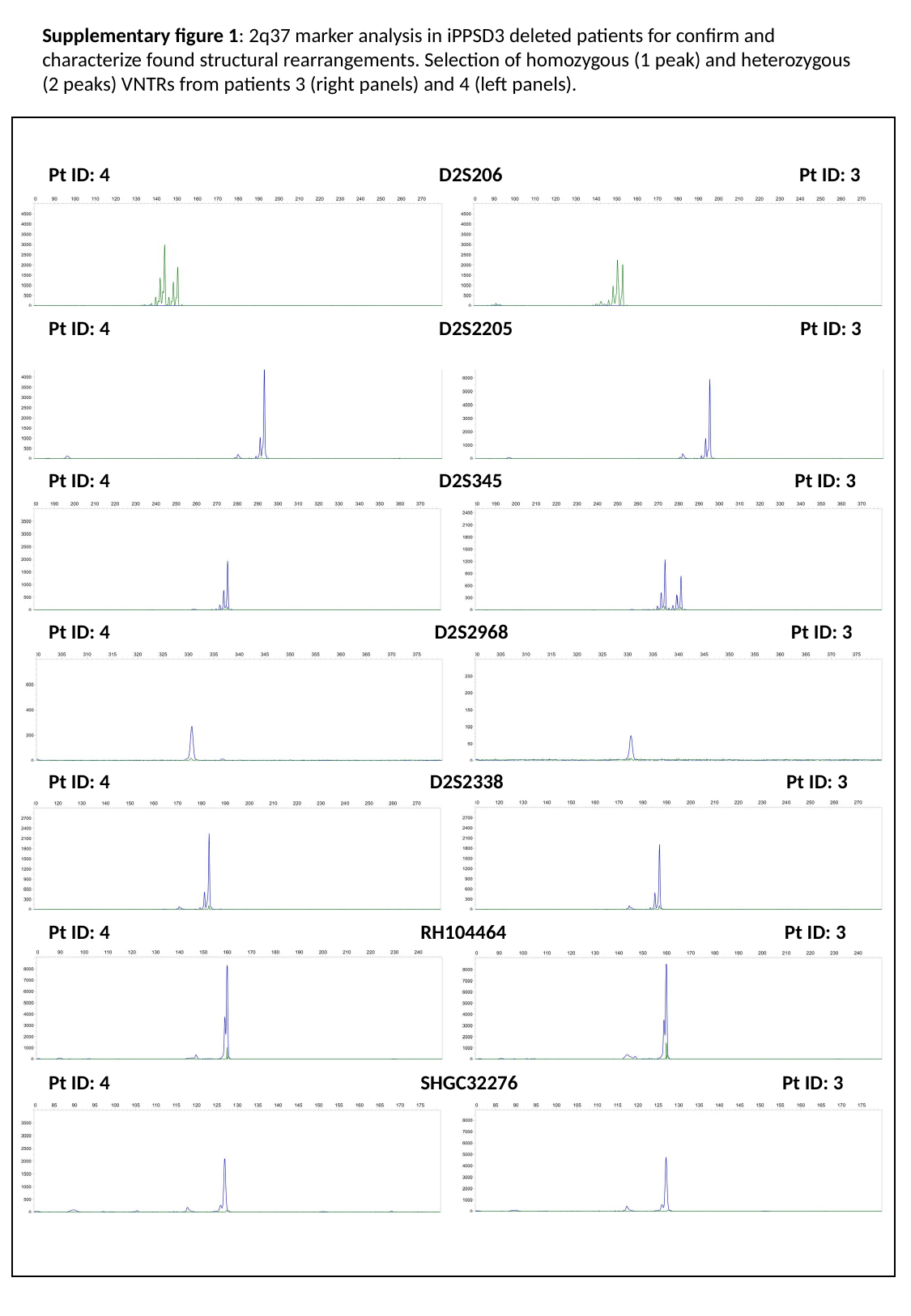

Supplementary figure 1: 2q37 marker analysis in iPPSD3 deleted patients for confirm and characterize found structural rearrangements. Selection of homozygous (1 peak) and heterozygous (2 peaks) VNTRs from patients 3 (right panels) and 4 (left panels).
 Pt ID: 4 D2S206 Pt ID: 3
 Pt ID: 4 D2S2205 Pt ID: 3
 Pt ID: 4 D2S345 Pt ID: 3
 Pt ID: 4 D2S2968 Pt ID: 3
 Pt ID: 4 D2S2338 Pt ID: 3
 Pt ID: 4 RH104464 Pt ID: 3
 Pt ID: 4 SHGC32276 Pt ID: 3
